# Dataset on social and economic indicators of fishing and non-fishing households from Natore, Bangladesh

**DOI:** 10.1016/j.dib.2026.113396

**Published:** 2026-11-03

**Authors:** Arijit Biswas Anirban, Md. Nayem Hossain, Md. Ashik Ali, Sumaiya Khatun, Istiak Ahammed, Priyosree Paul Chowdhury, Md Zubair Azam, Nakib Aman

**Affiliations:** a Department of Computer Science and Engineering, Pabna University of Science and Technology, Pabna, Bangladesh; b Department of Civil Engineering, Pabna University of Science and Technology, Pabna, Bangladesh; c Department of Social Work, Pabna University of Science and Technology, Pabna, Bangladesh; d Department of Computer Science and Engineering, Leading University, Sylhet, Bangladesh

**Keywords:** Wetland communities, Survey data, Rural households, Occupational classification, Food security, Health access, Migration, Machine learning benchmark

## Abstract

This data article presents quantitative socioeconomic and livelihood-related information collected from fishing and non-fishing households in the Chalan Beel region, Singra Upazila, Natore District, Bangladesh. A total of 282 household-level observations were gathered through face-to-face interviews during field visits, with an equal distribution of 141 fishing households and 141 non-fishing households. Data were collected using two structured questionnaires, one for each household group. Each questionnaire contained 59 questions under two broad sections: socioeconomic condition and livelihood diversification. The responses were recorded manually before being coded and processed in SPSS and Microsoft Excel. After coding and cleaning, the final dataset contains 77 variables derived from the questionnaire items.

The dataset covers demographic, educational, economic, housing, welfare, migration, food security, health, social participation, and livelihood diversification indicators. It provides raw and cleaned tabular data suitable for statistical analysis, cross-tabulation, comparative assessment, visualization, socioeconomic profiling, and construction of livelihood-related indices.

In addition to descriptive and comparative reuse, supervised machine-learning models were applied as a reproducible classification benchmark to illustrate one possible computational use of the survey data for distinguishing fishing and non-fishing households. The machine-learning workflow includes mixed-type preprocessing, categorical encoding, stratified cross-validation, reduced-feature evaluation, and final model testing. Researchers can reuse this dataset for comparative rural studies, policy modelling, food security analysis, health-access research, migration-related analysis, livelihood framework analysis, and machine-learning experiments. Development practitioners and policymakers may also use it to design targeted interventions for marginalized wetland communities.

Specifications Table

**Table:** 

Subject	Social Sciences
Specific subject area	Socioeconomic and livelihood diversification data of wetland-based rural households
Type of data	Table, Raw data, Cleaned data, Processed data, Metadata, Code, Model performance results
Data collection	Data were collected from 12 February 2026 to 27 February 2026 through structured questionnaires using face-to-face household interviews. A total of 282 households, including 141 fishing households and 141 non-fishing households, were surveyed in the Chalan Beel region, Singra Upazila, Natore District, Bangladesh. The responses were manually recorded, coded, and processed using Microsoft Excel and SPSS.
Data source location	Chalan Beel region, Singra Upazila, Natore District, Bangladesh.
Data accessibility	Repository name: Mendeley Data Data identification number: 10.17632/by8mht79xv.1 Direct URL to data: Household Socioeconomic and Livelihood Dataset of Fishermen and Non-Fishermen in Chalan Beel, Bangladesh
Related research article	None

## 1 Value of the Data

• These data provide a comparative household-level dataset from fishing and non-fishing households in the Chalan Beel region, Singra Upazila, Natore District, Bangladesh. The dataset is valuable because it covers two household groups from the same wetland context using similar survey domains, allowing researchers to compare socioeconomic and livelihood-related characteristics between the groups.
• The dataset contains 282 household observations and 77 variables derived from 59 structured questionnaire items after coding and processing in SPSS and Microsoft Excel. It includes information on education, employment, income, savings, housing, sanitation, drinking water, electricity, welfare access, migration, food security, health safety, women’s household participation, and livelihood diversification.
• Researchers can reuse the dataset for cross-tabulation, statistical analysis, socioeconomic profiling, livelihood vulnerability assessment, food security analysis, health-access studies, migration-related research, and construction of household-level socioeconomic or livelihood indices. The raw and cleaned tabular files also allow users to apply their own cleaning, coding, or analytical methods.
• The dataset is suitable for comparative studies across regions, occupations, or wetland-based communities. Other researchers can adapt the questionnaire structure, replicate the survey design in other rural or wetland ecosystems, or compare these data with future datasets to study livelihood change, rural development patterns, and access to public services.
• The dataset supports reproducible machine-learning classification benchmarks using structured household survey data. The included workflow can be reused to compare classification models, examine full-feature and reduced-feature settings, and evaluate preprocessing choices. These materials are provided as an example of computational reuse of the dataset rather than as evidence of measurement validity or internal consistency.
• Development practitioners and policymakers may use the dataset to support evidence-based planning for marginalized wetland communities. Its coverage of education, income stability, sanitation, health safety, welfare access, women’s participation, food security, and livelihood diversification can help identify areas for targeted development programs and community-level interventions.

## 2 Background

Chalan Beel is a major wetland system in Bangladesh where fisheries, agriculture, and seasonal livelihood activities are closely linked with rural household welfare. Ecological research has reported habitat degradation and biodiversity pressure in Chalan Beel, showing the value of structured data from this wetland context [1]. Small-scale fisheries are strongly connected with livelihood security, income generation, and resource-dependent rural development [2]. In Bangladesh, fisheries contribute to employment, food supply, and the wider rural economy [3]. Fishery-dependent households may also face vulnerability due to climate variability, resource uncertainty, and limited livelihood assets [4]. Livelihood diversification is important for fishing communities, although its outcomes can vary across social and economic settings [5]. Bangladesh-based research further links diversification with migration and non-migration decisions in environmentally sensitive communities [6]. Value-chain studies also show that fishing households may face unequal bargaining power and limited influence over livelihood-related decisions [7]. However, comparable household-level data covering both fishing and non-fishing households from the same wetland setting remain limited. Therefore, this dataset was prepared from the Chalan Beel region, Singra Upazila, Natore District, Bangladesh, to document socioeconomic, livelihood, food security, health, migration, welfare, and community participation indicators for comparative analysis and to provide a reproducible example of machine-learning classification using the released data.

## 3 Data Description

The dataset consists of a curated household-level socioeconomic and livelihood survey dataset collected from the Chalan Beel area of Bangladesh. It contains records from 282 households representing two household groups: fishing and non-fishing households. The dataset is balanced, with 141 fishing households and 141 non-fishing households. The main cleaned dataset includes 77 original questionnaire-based variables, beginning with the household identifier id and ending with cultural_participation. The target variable is community, which indicates whether each household belongs to the fishing or non-fishing household group.

The dataset is structured to support direct reuse in socioeconomic description, livelihood comparison, metadata-based interpretation, and reproducible machine-learning classification experiments. The questionnaire instruments include parallel sections on socioeconomic condition and livelihood diversification for both household groups. These sections cover education, employment, income, housing, land ownership, welfare access, livelihood diversification, migration, food security, health, treatment choice, community participation, and cultural participation. Some questionnaire items are occupation-specific, such as fishing net or boat ownership and fish-farming training in the questionnaire for fishing households, and cultivation land ownership and agricultural training in the questionnaire for non-fishing households. Therefore, the cleaned dataset uses neutral variable descriptions where required to support comparison between the two household groups. The overall structure of the dataset and accompanying repository files is shown in Fig. 1.

**Fig. 1:**
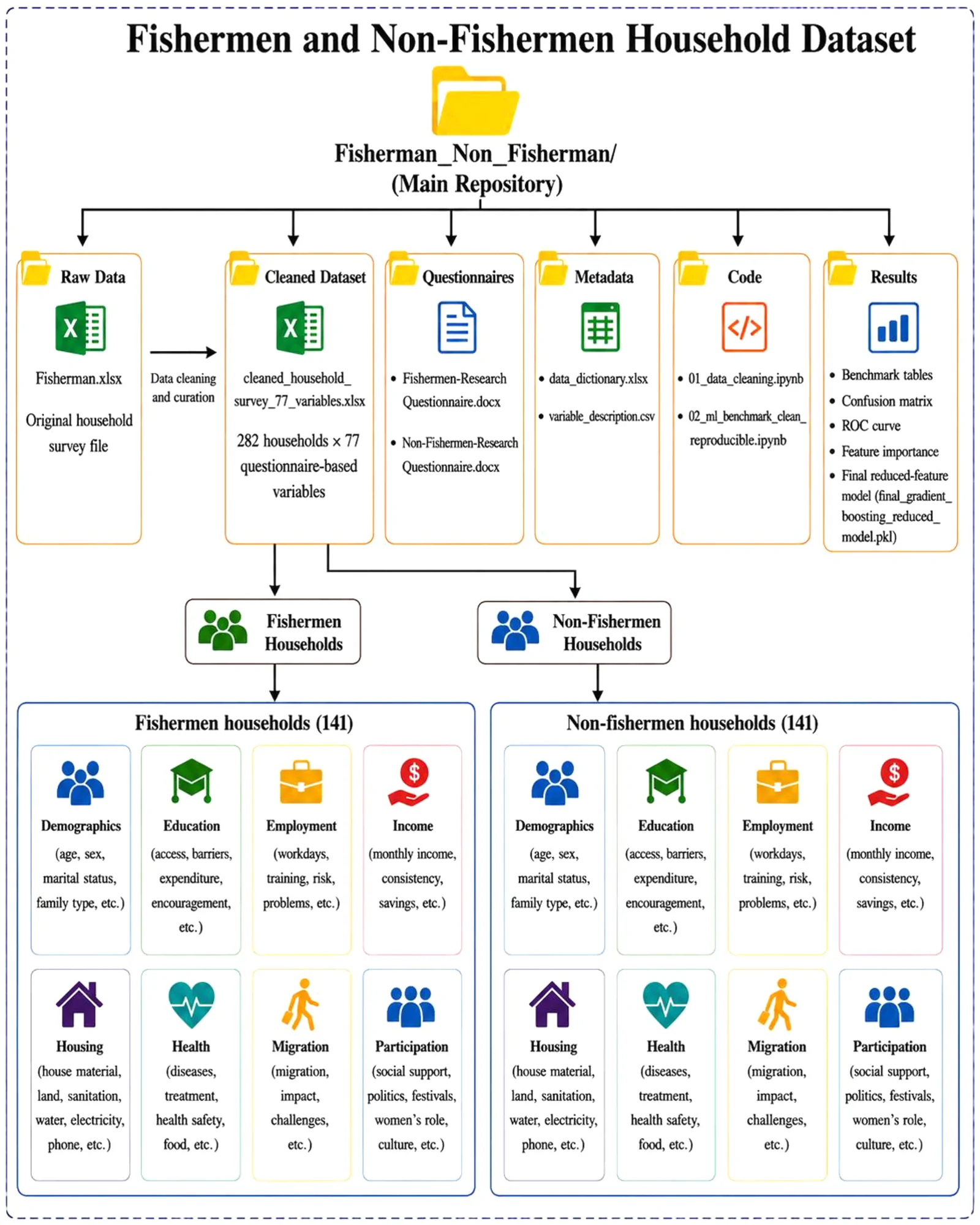
Structure of the main dataset.

The overall organization of the dataset and associated repository files is presented in Fig. 1. The repository separates the raw survey file, cleaned household-level dataset, questionnaire instruments, metadata files, reproducible notebooks, and machine-learning benchmark outputs. The cleaned dataset contains 282 household records and 77 original questionnaire-based variables, with equal representation of fishing and non-fishing households. The class-wise structure is shown to clarify that the dataset is balanced and suitable for comparative household-level analysis and reproducible classification benchmarks.

The dataset variables were derived from the two structured questionnaire instruments used for fishing and non-fishing households. Both questionnaires contain sections on socioeconomic condition and livelihood diversification, including household composition, education, employment, income, housing, social support, welfare access, women’s participation, migration, food security, health, treatment choice, and community participation. The questionnaire for fishing households includes occupation-specific items such as fishing net or boat ownership, fish-farming training, eco-friendly fishing, and loans for fishing assets, while the questionnaire for non-fishing households includes parallel items on cultivation land, agricultural training, eco-friendly agriculture, and loans for agriculture or other work.

To maintain comparability between the two household groups, the cleaned dataset uses harmonized variable names and neutral descriptions for occupation-specific responses. For example, fishing net or boat ownership and cultivation land ownership are represented as occupation-related productive assets, while fish-farming training and agricultural training are treated as occupation-specific training. This structure allows the dataset to retain group-specific livelihood information while supporting household-level comparison between fishing and non-fishing households. The main survey domains and representative variables are summarized in Table 1.

**Table 1:** Survey domains and representative variables included in the curated household-level dataset.

Survey domain	Description	Representative variables
Demographic characteristics	Household demographic profile and family composition	age, sex, marital_status, family_members, family_type
Education	Education access, educational participation, barriers, expenditure, and encouragement for higher education	household_head_education, children_education_chance, members_in_education, education_financial_limitation, child_education_spending, higher_education_encouragement
Employment and occupational condition	Working days, occupational training, workplace risk, and work-related problems	working_days_month, occupational_training, work_risk, natural_disaster, piracy, health_problem
Economy and income	Monthly income, additional income, income consistency, and savings	monthly_income, income_beyond_primary, income_consistency, savings
Housing and living conditions	Housing material, phone use, land ownership, sanitation, drinking water, and electricity access	house_building_material, phone_use, living_land, total_land, sanitation_type, water_source, electricity
Family, social support, and welfare	Family crisis support, community organization, political involvement, social participation, and welfare access	family_crisis_cooperation, community_association, local_politics_involvement, social_festival_participation, gov_allowance_receiving, welfare_access_ease
Women’s participation	Women’s role in household decisions, income engagement, and financial access	women_household_decisions, women_income_engagement, women_equal_financial_access
Livelihood diversification	Alternative income, diversification interest, additional family work, productive assets, training, and loans	alternative_income, alternative_income_type, occupation_diversification_interest, family_extra_income_work, own_land_boat_net, working_training, loan_taking, loan_source
Migration and social mobility	Work-related migration, migration impact, and migration challenges	migrated_for_work, migration_impact, migration_challenge
Food security and health	Food scarcity, seasonal food availability, food expenditure, health safety, common diseases, and treatment choice	food_scarcity_experience, seasonal_food_availability, income_spent_on_food, health_safety, fever_cold, diarrhoea, gastric, village_doctor, govt_hospital, mbbs_doctor
Community and cultural participation	Community development role, entertainment sources, social or religious participation, and cultural participation	community_development_role, tv, radio, social_religious_participation, cultural_participation

The cleaned dataset contains a balanced binary household-group structure. Each household record is assigned to one of two classes using the target variable community. The dataset includes 141 fishing households and 141 non-fishing households, resulting in a total of 282 household observations. This balanced structure supports comparative household-level analysis and supervised classification experiments without class imbalance at the target-variable level. Key descriptive characteristics of the achieved sample are summarized in Table 2.

**Table 2:** Descriptive characteristics of the surveyed households, overall and by household group.

Characteristic	Overall (n=282)	Fishing households (n=141)	Non-fishing households (n=141)
Respondent age group, n (%)			
20-30 years	22 (7.8%)	9 (6.4%)	13 (9.2%)
31-35 years	63 (22.3%)	29 (20.6%)	34 (24.1%)
36-40 years	93 (33.0%)	44 (31.2%)	49 (34.8%)
41-45 years	54 (19.1%)	23 (16.3%)	31 (22.0%)
46-50 years	24 (8.5%)	17 (12.1%)	7 (5.0%)
51-60 years	19 (6.7%)	12 (8.5%)	7 (5.0%)
61-70 years	7 (2.5%)	7 (5.0%)	0 (0.0%)
Respondent sex, n (%)			
Male	274 (97.2%)	136 (96.5%)	138 (97.9%)
Female	8 (2.8%)	5 (3.5%)	3 (2.1%)
Respondent marital status: Married, n (%)	282 (100.0%)	141 (100.0%)	141 (100.0%)
Household size, mean ± SD	4.37 ± 1.09	4.56 ± 1.17	4.17 ± 0.97
Household head education, n (%)			
Illiterate	98 (34.8%)	58 (41.1%)	40 (28.4%)
Primary	87 (30.9%)	48 (34.0%)	39 (27.7%)
Secondary	58 (20.6%)	22 (15.6%)	36 (25.5%)
Higher secondary	25 (8.9%)	12 (8.5%)	13 (9.2%)
Honors or above	14 (5.0%)	1 (0.7%)	13 (9.2%)
Monthly household income, median (IQR), BDT	17,000 (15,000-20,000)	17,000 (15,000-18,000)	18,000 (15,000-22,000)
Household savings: Yes, n (%)	77 (27.3%)	18 (12.8%)	59 (41.8%)

Table 2 provides a brief descriptive profile of the achieved sample. All 282 respondents reported being married. Marital status was not used as an eligibility criterion for the survey; rather, this was an observed characteristic of the respondents who completed the interviews. The household head was the preferred respondent, while in eight households the spouse completed the interview because the household head remained unavailable during the return visit.

The cleaned dataset was further documented using a variable-level data dictionary to support clear interpretation and reuse. Table 3 presents all 77 variables included in the cleaned household survey dataset, together with their descriptions, data types, possible values, missing-value counts, and unique-value counts. This table helps users understand how the original questionnaire responses were converted into machine-readable columns and supports appropriate variable selection for statistical analysis, comparative assessment, and reproducible machine-learning experiments.

**Table 3:** Variable-level data dictionary of the cleaned household survey dataset.

Column name	Description	Type	Possible values	Missing values	Unique values
Id	Unique household identification number	Identifier	Unique ID	0	282
community	Household livelihood group	Categorical	Fishermen; Non-fishermen	0	2
Age	Age group of respondent	Ordinal categorical	20-30 Years; 31-35 Years; 36-40 Years; 41-45 Years; 46-50 Years; 51-60 Years; 61-70 Years	0	7
Sex	Sex or gender identity of respondent	Categorical	Female; Male	0	2
marital_status	Current marital status of respondent	Categorical	Married	0	1
family_members	Number of members in the household	Numerical	1; 2; 3; 4; 5; 6; 7; 9	0	8
family_type	Type of household family structure	Categorical	Joint Family; Unit Family	0	2
household_head_education	Highest education level of household head	Ordinal categorical	Higher secondary; Honors or above; Illiterate; Primary; Secondary	0	5
children_education_chance	Access of household members to education	Ordinal categorical	No; Partially; Yes, enough	0	3
members_in_education	Number of household members currently studying	Numerical	0; 1; 2; 3	0	4
education_financial_limitation	Financial limitation as a barrier to education	Categorical	No; Yes	0	2
education_unawareness	Lack of awareness as a barrier to education	Categorical	No; Yes	0	2
family_illiteracy	Family illiteracy as a barrier to education	Categorical	No; Yes	0	2
education_other_challenges	Other reported barriers to education	Categorical	No; Yes	0	2
child_education_spending	Annual household expenditure on child education	Numerical	0; 10000; 12000; 14000; 15000; 16000; 18000; 20000; 22000; 24000; 25000; 30000; 32000; 35000; 40000; 45000; 5000; 50000; 6000; 60000; 7000; 70000; 8000; 80000; 9000	0	25
higher_education_encouragement	Family encouragement for higher education	Categorical	No; Yes	0	2
why_not_encourage	Reason for not encouraging higher education	Categorical	Child not interested or poor performance; Lack of employment opportunities; Not applicable; Unable to afford expenses; Unaware about higher education	0	5
working_days_month	Number of working days per month	Ordinal categorical	15-20 days; 20-26 days; 26-30 days; Less than 15 days	0	4
occupational_training	Whether respondent received occupational training	Categorical	No; Yes	0	2
work_risk	Perceived level of workplace risk	Ordinal categorical	A bit risky; High risk; No risk	0	3
natural_disaster	Natural disaster reported as a workplace problem	Categorical	No; Yes	0	2
piracy	Piracy reported as a workplace problem	Categorical	No; Yes	0	2
health_problem	Health issue reported as a workplace problem	Categorical	No; Yes	0	2
monthly_income	Average monthly household income	Numerical	10000; 12000; 13000; 14000; 15000; 16000; 17000; 18000; 20000; 22000; 24000; 25000; 26000; 27000; 3000; 30000; 35000; 7000; 8000	0	19
income_beyond_primary	Whether household has income beyond primary occupation	Categorical	No; Yes	0	2
income_consistency	Stability of household income	Ordinal categorical	Partially stable; Stable; Unstable	0	3
savings	Household savings practice	Categorical	No; Yes	0	2
house_building_material	Main construction material of household dwelling	Categorical	Bamboo/Wood; Brick; Soil; Tin	0	4
phone_use	Type of phone used by household	Categorical	Button Phone; Smartphone	0	2
living_land	Whether household lives on its own land	Categorical	No; Yes	0	2
if_no_living_land	Housing arrangement if household does not own living land	Categorical	Not applicable; State-owned land	0	2
total_land	Total land owned by household	Numerical	0.0; 1.5; 10.0; 12.0; 13.0; 13.2; 132.0; 15.0; 150.0; 16.5; 165.0; 17.0; 2.0; 20.0; 231.0; 264.0; 3.0; 33.0; 330.0; 4.0; 40.0; 42.0; 49.5; 495.0; 5.0; 50.0; 58.0; 6.0; 60.0; 66.0; 7.0; 70.0; 75.0; 8.0; 8.25; 9.0; 99.0	0	37
sanitation_type	Type of household sanitation facility	Categorical	Kaccha; Sanitary	0	2
water_source	Main source of household drinking water	Categorical	Pond/river; Tubewell	0	2
electricity	Household access to electricity	Categorical	No; Yes	0	2
family_crisis_cooperation	Family support during household crisis	Ordinal categorical	Always cooperative; Less cooperative; Never; Sometimes cooperative	0	4
community_association	Presence of community association or social organization	Categorical	No; Yes	0	2
local_politics_involvement	Involvement in local politics	Categorical	No; Yes	0	2
social_festival_participation	Participation in social festivals or events	Ordinal categorical	Always; Never; Sometimes	0	3
women_household_decisions	Women’s participation in household decision-making	Categorical	No; Yes	0	2
women_income_engagement	Women’s engagement in income-generating activities	Categorical	No; Yes	0	2
women_equal_financial_access	Women’s equal access to financial resources	Categorical	No; Yes	0	2
gov_allowance_receiving	Receipt of government allowance or welfare support	Categorical	No; Yes	0	2
welfare_access_ease	Ease of access to government or NGO welfare programs	Ordinal categorical	Difficult; Somewhat Easy; Very Difficult; Very Easy	0	4
alternative_income	Availability of alternative household income source	Categorical	Have sources; No source	0	2
alternative_income_type	Type of alternative income source	Categorical	Cattle Farming; Crop Threshing; Dates Jaggery Production; Duck Farming; Farming; Grocery shop and Business; No alternative income	0	7
occupation_diversification_interest	Interest in livelihood diversification	Categorical	No; Yes	0	2
occupation_diversification_impact	Perceived economic impact of livelihood diversification	Ordinal categorical	Extreme; Less; Medium	0	3
family_extra_income_work	Whether family members work for additional income	Categorical	No; Yes	0	2
own_land_boat_net	Ownership of occupation-related productive asset, such as fishing net/boat for fishing households or cultivation land for non-fishing households	Categorical	No; Yes	0	2
income_beyond_season	Main income source beyond seasonal, fishing, farming, or agricultural work	Categorical	Business; Farming; Labor; Others; Rickshaw van puller	0	5
working_training	Occupation-specific training or technical assistance for fishing, fish farming, agriculture, or related work	Categorical	No; Yes	0	2
loan_taking	Whether the household has taken loan or financial assistance for occupation-related work	Categorical	No; Yes	0	2
loan_source	Source of loan or financial assistance for occupation-related work	Categorical	Loan from Moneylender; NGOs; No loan; Private Commercial Banks; Unknown	0	5
migrated_for_work	Whether household member migrated for work	Categorical	No; Yes	0	2
migration_impact	Perceived effect of migration on household income	Categorical	Negative impact; No impact; Positive impact	0	3
migration_challenge	Main challenge faced during migration	Categorical	High migration costs; Lack of employment opportunities; Other; Unstable living conditions	0	4
food_scarcity_experience	Experience of household food scarcity	Categorical	No; Yes	0	2
seasonal_food_availability	Seasonal variation in household food availability	Categorical	No; Yes	0	2
income_spent_on_food	Share of household income spent on food	Ordinal categorical	25-50%; 50-75%; Less than 25%; More than 75%	0	4
health_safety	Perceived workplace health and safety condition	Ordinal categorical	Average; Satisfactory; Unsatisfied	0	3
fever_cold	Fever or cold as a common health issue	Categorical	No; Yes	0	2
diarrhoea	Diarrhoea as a common health issue	Categorical	No; Yes	0	2
gastric	Gastric problem as a common health issue	Categorical	No; Yes	0	2
headache	Headache as a common health issue	Categorical	No; Yes	0	2
malnutrition	Malnutrition as a common health issue	Categorical	No; Yes	0	2
other_diseases	Other reported diseases or health issues	Categorical	No; Yes	0	2
village_doctor	Use of village doctor for treatment	Categorical	No; Yes	0	2
govt_hospital	Use of government hospital for treatment	Categorical	No; Yes	0	2
mbbs_doctor	Use of MBBS doctor for treatment	Categorical	No; Yes	0	2
kobiraj	Use of traditional healer or Kobiraj for treatment	Categorical	No; Yes	0	2
health_safety_steps	Measures taken to ensure health and safety at the workplace	Categorical	No; Yes	0	2
community_development_role	Perceived contribution to community development	Categorical	Extreme; No role; Somewhat	0	3
tv	Television as a source of entertainment or information	Categorical	No; Yes	0	2
radio	Radio as a source of entertainment or information	Categorical	No; Yes	0	2
social_religious_participation	Participation in social or religious events	Categorical	No; Yes	0	2
cultural_participation	Participation in cultural events	Categorical	No; Yes	0	2

The main cleaned dataset is provided as cleaned_household_survey_77_variables.xlsx inside the data/cleaned/ folder. The first column, id, is an identifier variable and is not intended to be used as a predictive feature. The target variable, community, identifies the household group. The remaining variables describe household-level socioeconomic, livelihood, health, migration, food-security, and participation-related characteristics. Although several variables appear numeric in meaning, most variables are categorical or ordinal categorical because they were recorded as survey-response categories. For example, age is stored as ordered age groups rather than as a continuous numerical variable.

The dataset repository is organized into separate folders for raw data, cleaned data, metadata, questionnaires, code, and machine-learning results. This structure separates the original survey file from the curated analytical dataset and keeps documentation, reproducible notebooks, and benchmark outputs in independent folders. The file-wise organization of the repository is summarized in Table 4.

**Table 4:** Repository files and descriptions provided with the dataset.

Folder	File	Description
data/raw/	Fisherman.xlsx	Original raw household survey file before cleaning and curation
data/cleaned/	cleaned_household_survey_77_variables.xlsx	Final cleaned dataset containing 282 household observations and 77 original questionnaire-based variables
questionnaires/	Fishermen-Research Questionnaire.docx	Structured questionnaire used for fishing households
questionnaires/	Non-Fishermen-Research Questionnaire.docx	Structured questionnaire used for non-fishing households
metadata/	data_dictionary.xlsx	Detailed data dictionary containing variable names, descriptions, types, possible values, missing-value information, and unique values
metadata/	variable_description.csv	Lightweight variable description file containing variable name, description, type, and possible values
code/	01_data_cleaning.ipynb	Reproducible notebook used for data cleaning, variable harmonization, and preparation of the final cleaned dataset
code/	02_ml_benchmark_clean_reproducible.ipynb	Reproducible notebook used for machine-learning classification benchmark experiments
results/	ml_benchmark_results_full_features.csv/.xlsx	Cross-validation benchmark results using the full predictor set
results/	ml_benchmark_results_reduced_features.csv/.xlsx	Cross-validation benchmark results using the reduced predictor set
results/	ml_benchmark_full_vs_reduced_comparison.csv/.xlsx	Comparison between full-feature and reduced-feature benchmark results
results/	classification_report_gradient_boosting_reduced.csv/.xlsx	Classification report for the final reduced-feature Gradient Boosting model
results/	confusion_matrix_reduced_gradient_boosting.csv/.xlsx/.png/.pdf	Confusion matrix outputs for the final reduced-feature Gradient Boosting model
results/	roc_curve_gradient_boosting_reduced.png/.pdf	ROC curve output for the final reduced-feature Gradient Boosting model
results/	gradient_boosting_feature_importance_full_features.csv/.xlsx	Feature-importance values from the full-feature Gradient Boosting model
results/	gradient_boosting_feature_importance_reduced_features.csv/.xlsx	Feature-importance values from the reduced-feature Gradient Boosting model
results/	top20_feature_importance_reduced_gradient_boosting_clean.png/.pdf	Figure showing the top 20 feature-importance values from the reduced-feature Gradient Boosting model
results/	final_gradient_boosting_reduced_model.pkl	Final saved reduced-feature Gradient Boosting benchmark model
results/	final_test_result_gradient_boosting_reduced.csv/.xlsx	Independent test-set performance results for the final reduced-feature Gradient Boosting model

The metadata files document the cleaned dataset for reuse. The detailed data dictionary records variable names, descriptions, types, possible values, missing-value status, and unique values. The shorter variable-description file provides a concise reference for variable name, description, type, and possible values. The dataset includes identifier, numerical, categorical, and ordinal categorical variables; id is an identifier, and community is the grouping variable.

The repository also includes benchmark outputs generated from the cleaned dataset, including result tables, classification reports, confusion matrices, ROC curves, feature-importance files, final test-set results, and the saved reduced-feature Gradient Boosting model. These files are provided as supplementary benchmark and reproducibility outputs for users who wish to inspect or reproduce the classification experiments.

## 4 Experimental Design, Materials and Methods

The study was conducted in the Chalan Beel region, Singra Upazila, Natore District, Bangladesh. Chalan Beel is one of the important wetland ecosystems of Bangladesh and is well known for its fisheries, agriculture, and wetland-dependent rural livelihoods. Households in this region depend on a combination of fishing, farming, seasonal labour, and other income-generating activities. Because of this mixed livelihood structure, the area provides an appropriate setting for comparing fishing and non-fishing households.

Singra Upazila of Natore District was selected as the study area because it represents an important part of the Chalan Beel wetland system and includes both fishing and non-fishing households living under similar ecological and rural conditions. The area is suitable for examining household-level socioeconomic characteristics, livelihood diversification, food security, health, migration, and access to public services. The geographic context of the study area is presented through two location maps. One map shows the position of Natore District within Bangladesh, and the other shows the location of Singra Upazila within the district. These maps help readers understand the spatial setting of the survey and the wetland-based context of the dataset. Fig. 2 shows the two location maps used to present the study area: the Bangladesh map indicating Natore District and the Singra Upazila map showing the local survey setting.

**Fig. 2:**
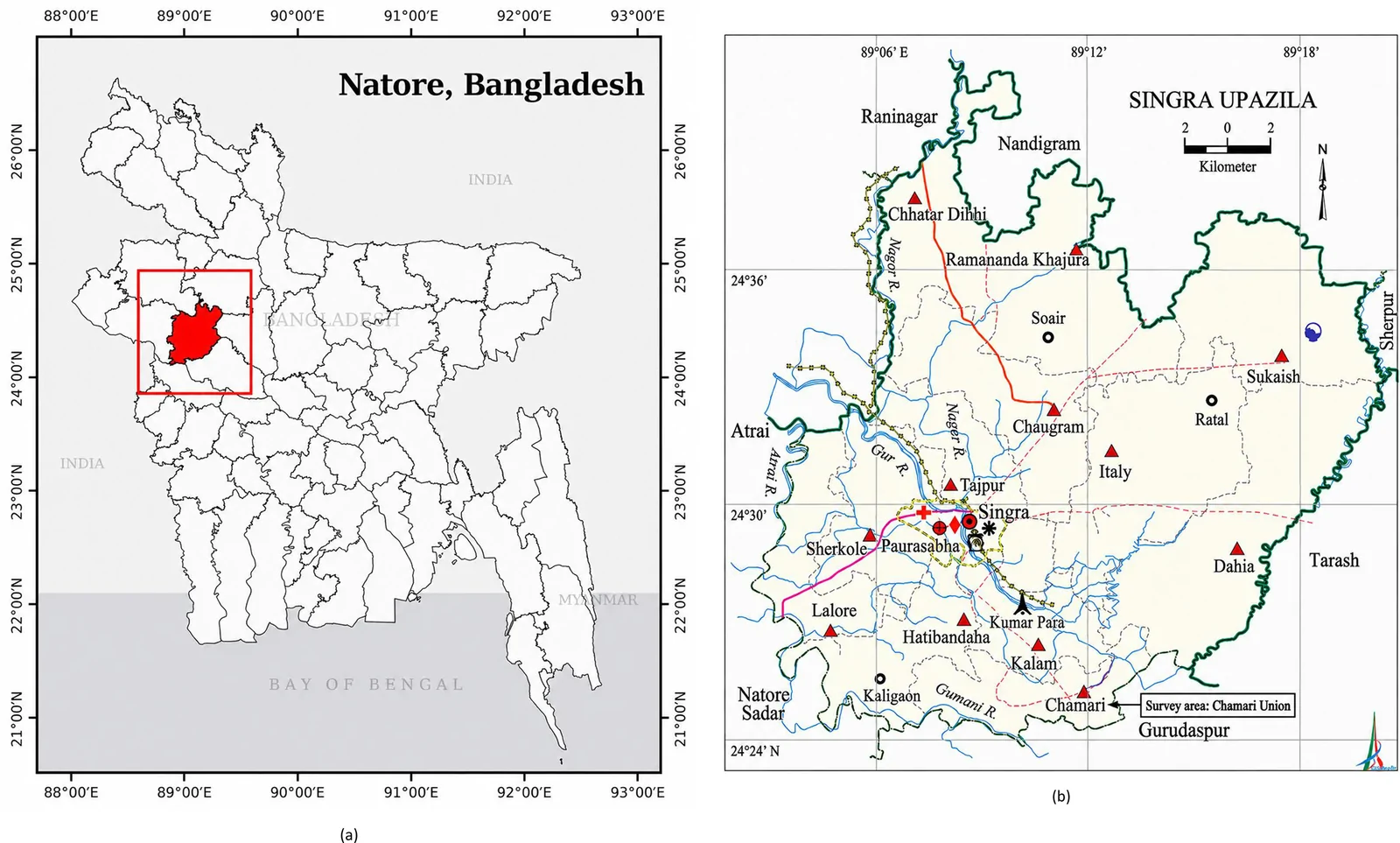
Location of the study area: (a) Natore District within Bangladesh and (b) Singra Upazila within Natore District, showing Chamari Union, where the household survey was conducted.

Fig. 3 summarizes the sequential workflow followed to prepare, document, and release the dataset. The workflow includes questionnaire development, sampling, household survey data collection, raw data compilation, data cleaning, logical missing-value handling, cleaned dataset preparation, metadata documentation, a machine-learning classification benchmark, and repository organization. This workflow was designed to support transparent dataset preparation, reproducibility, and reuse across descriptive, comparative, and computational analyses.

**Fig. 3:**
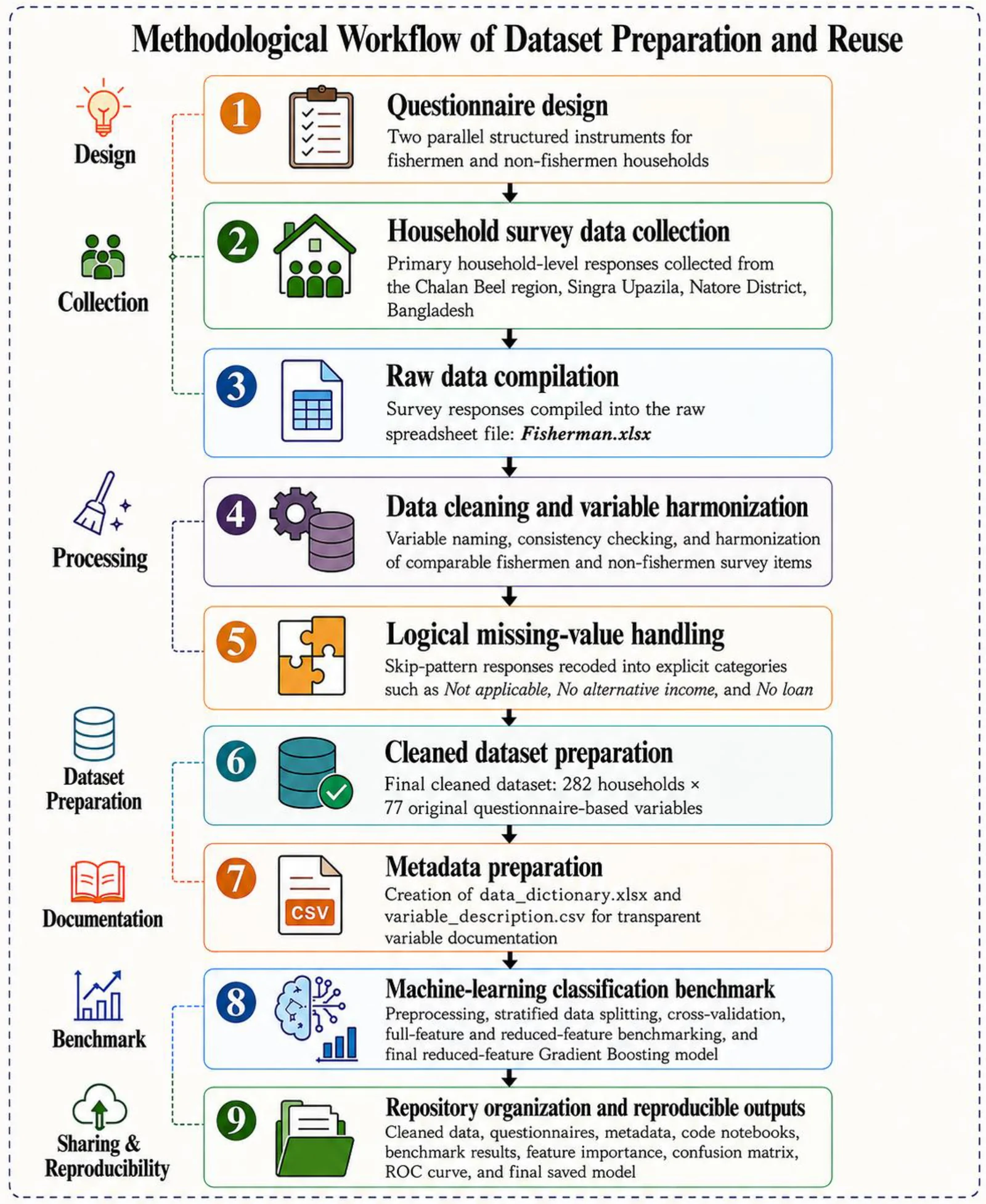
Methodological workflow of dataset preparation, documentation, and computational reuse.

### 4.1 Sampling procedure

The study area, Chamari Union in Singra Upazila, was purposively selected because fishing-dependent households are relatively common in this wetland-associated area. A household list for the study area was obtained from Chamari Union Parishad, which reported a total of 1,056 households. The administrative household list did not contain a prior classification of households as fishing or non-fishing. Before sampling, households were screened according to their primary livelihood and divided into two strata: fishing households and non-fishing households. A household was classified as a fishing household when the household head’s primary occupation was fishing and the household depended predominantly on fishing as its main livelihood source. Small supplementary activities, such as occasional farming for household consumption or small-scale cattle or duck rearing, did not alter this classification when fishing remained the dominant livelihood. Non-fishing households were those whose household head and primary livelihood were not connected to fishing. Households were then randomly selected within each stratum using a lottery method. The household survey was conducted from 12 February 2026 to 27 February 2026 after receiving departmental permission and local field-level permission for data collection.

The sample size was calculated using Cochran’s formula with a 95% confidence level and 5% margin of error.

Here,

▪ $n_{0}=\text{Initial}\;\text{sample}\;\text{size},$
▪ z=confidencelevelat95%=1.96,
▪ p=assumedpopulationproportion=0.5(thismaximizestherequiredsamplesize),
▪ q=1−p,
▪ e=errorlimitsof5%(0.05).
  $$n_{0}=\;\frac{z^{2}\;\times \;p\;\times \;q}{e^{2}}\;=\;\frac{{\left(1.96\right)}^{2}\;\times \;0.5\;\times \;0.5}{{\left(0.05\right)}^{2}}\;=\;\frac{0.9604}{0.0025}\;=384$$
▪ n=Samplesize,
▪ $n_{0}=384\;\left(\text{Initial}\;\text{sample}\;\text{size}\right),$
▪ N=1056(targetpopulationsizeinhouseholds)
  $$n=\;\frac{n_{0}}{1+\;\left\{\frac{n_{0}-1}{N}\right\}}\;=\;\frac{384}{1+\;\left\{\frac{384-1}{1056}\right\}}\;=\;\frac{384}{1.363}\;=281.73\;\approx 282$$

Accordingly, the final sample size was determined to be 282 households. Equal allocation was intentionally used across the two strata, with 141 fishing households and 141 non-fishing households, to support balanced comparison between the groups rather than to reproduce the underlying occupational composition of the source population. All 282 selected households completed the survey. No household refused participation, and no selected household was replaced. The household head was the preferred respondent for each selected household because the questionnaire focused mainly on household livelihood and socioeconomic conditions. If the household head was unavailable during the first visit, the team returned on a later day. When the household head was still unavailable during the return visit, the spouse was interviewed if they were familiar with the household information required for the survey. In eight households, the spouse provided the interview. Because the 141/141 allocation was not designed to reflect the population proportions of fishing and non-fishing households, pooled unweighted estimates from this balanced sample should not be interpreted as population-level prevalence estimates for Chamari Union.

### 4.2 Questionnaire design and survey structure

The study used two structured questionnaires, prepared separately for fishing and non-fishing households. The questionnaires were developed specifically for this study based on the research objectives and ideas drawn from relevant published studies on socioeconomic conditions and livelihood diversification, rather than being adopted from a single established instrument. The initial questions were reviewed by the field team’s supervisor from the Department of Social Work, and necessary revisions were made before the main survey. The questionnaires were then pre-tested with a small group of people similar to the target respondents. Based on the feedback from this pre-test, minor changes were made to the wording and structure of selected questions to improve clarity.

Each questionnaire contained two main sections. The first section focused on socioeconomic condition and included items on demographic characteristics, family structure, education, employment, workplace risk, income, savings, housing condition, land ownership, sanitation, drinking water, electricity access, family support, community association, social participation, women’s household and economic participation, and welfare-program access. The second section focused on livelihood diversification and included items on alternative income sources, livelihood diversification interest, additional family income activities, productive assets, training, loans, migration, food scarcity, seasonal food availability, food expenditure, health and safety, common diseases, treatment choices, community development role, and social or cultural participation.

The two questionnaires were mostly parallel, but selected items were adapted to the livelihood context of each group. The questionnaire for fishing households included occupation-specific questions on fishing net or boat ownership, fish-farming training, eco-friendly fishing, and loans for fishing nets or boats. The questionnaire for non-fishing households included parallel questions on cultivation land, agricultural training, eco-friendly agriculture, and loans for agriculture or other work. This design allowed the final dataset to preserve occupation-specific survey information while maintaining a harmonized structure for comparison between fishing and non-fishing households.

### 4.3 Questionnaire administration and field procedure

The questionnaires were written in English, but all face-to-face interviews were conducted in Bangla. A separate formally translated or back-translated Bangla version was not prepared. Before fieldwork, the team discussed the meaning of the questions and agreed on a common way of explaining them in Bangla to keep the interviews as consistent as possible. A few members of the field team had previous experience with survey and field-data collection through academic coursework and relevant workshops, and they briefed the other team members before the survey. The household head was the preferred respondent for each selected household because the questionnaire focused mainly on household livelihood and socioeconomic conditions. If the household head was unavailable during the first visit, the team returned on a later day. When the household head was still unavailable during the return visit, the spouse was interviewed if they were familiar with the household information required for the survey. In eight cases, the spouse completed the interview. Interviews generally took about 25-30 minutes. No financial or material incentive was provided to respondents, and all selected households ultimately completed the survey. Each completed interview contributed one household-level observation to the dataset; the term “respondent” refers to the individual who answered the questionnaire on behalf of the selected household.

### 4.4 Data cleaning and variable harmonization

The raw survey responses were first compiled into a spreadsheet format and then reviewed for consistency, completeness, and variable structure. The raw data file was stored as Fisherman.xlsx, while the final cleaned dataset was saved as cleaned_household_survey_77_variables.xlsx. During cleaning, the dataset was organized into a consistent household-level format, where each row represents one household and each column represents one questionnaire-derived variable.

A key step in the cleaning process was the harmonization of variables between the questionnaires for fishing and non-fishing households. Although the two questionnaires followed a largely parallel structure, some livelihood-related questions were worded differently because of the occupational context of each group. For example, the questionnaire for fishing households asked about fishing net or boat ownership, fish-farming training, eco-friendly fishing, and loans for fishing nets or boats. The questionnaire for non-fishing households used parallel questions related to cultivation land, agricultural training, eco-friendly agriculture, and loans for agriculture or other work.

To combine the two questionnaires into one comparable dataset, occupation-specific responses were converted into harmonized variable names and neutral descriptions. For example, fishing net or boat ownership and cultivation land ownership were represented under the broader idea of occupation-related productive assets. Similarly, fish-farming training and agricultural training were treated as occupation-specific training. This approach allowed the dataset to preserve the original livelihood meaning of each response while making the final variables suitable for comparison between fishing and non-fishing households.

The cleaned dataset retained 77 original questionnaire-based variables. The first column, id, was kept as a household identifier, while community was used as the grouping or target variable. The remaining variables describe household demographic characteristics, education, employment, income, housing, welfare access, livelihood diversification, migration, food security, health, treatment choice, and community participation. No derived scoring variables were included in the main cleaned dataset, so the dataset remains directly connected to the original questionnaire structure.

### 4.5 Logical missing-value handling and variable typing

The cleaned dataset was prepared to avoid unresolved questionnaire-based missing values. In the raw survey file, some missing entries resulted from questionnaire skip patterns rather than from non-response or data-entry error. For example, when a household reported living on its own land, the follow-up question about housing arrangement without own land was not applicable. Similarly, when a household reported no alternative income source or no loan, the follow-up questions on alternative income type or loan source were logically not applicable.

For this reason, logical missing values were converted into explicit response categories during data cleaning. This step made the final dataset easier to interpret and prevented valid skip-pattern responses from being treated as missing information. The main logical missing-value corrections are shown in Table 5.

**Table 5:** Logical missing-value handling in the cleaned dataset.

Variable	Logical interpretation of missing value	Recoded value
if_no_living_land	Missing value indicates that the household lives on its own land	Not applicable
why_not_encourage	Missing value indicates that higher education is encouraged in the household	Not applicable
alternative_income_type	Missing value indicates that the household has no alternative income source	No alternative income
loan_source	Missing value indicates that the household did not take a loan	No loan

After these corrections, the final cleaned dataset contained no missing values. This cleaning decision preserved the original questionnaire logic while making the dataset suitable for descriptive analysis, metadata documentation, and reproducible machine-learning experiments.

Variables were also reviewed according to their actual response format rather than only their apparent meaning. The dataset contains identifier, numerical, categorical, and ordinal categorical variables. The id column was retained only as a household identifier, while community was used as the target or grouping variable. Only five variables were treated as numerical in the machine-learning workflow: family_members, members_in_education, child_education_spending, monthly_income, and total_land.

Other variables were treated as categorical or ordinal categorical because they were recorded as response categories. For example, age was not recorded as exact age in years in the final cleaned dataset; it was recorded as ordered age ranges such as 20–30 Years, 31–35 Years, 36–40 Years, 41–45 Years, 46–50 Years, 51–60 Years, and 61–70 Years. Therefore, age should be interpreted as an ordinal categorical variable rather than as a continuous numerical variable.

### 4.6 Machine-learning benchmark design

A machine-learning classification benchmark was prepared as an example of computational reuse of the cleaned household dataset. The aim was to document a reproducible supervised-learning workflow for distinguishing fishing and non-fishing households under the reported feature settings. The benchmark was not intended to validate the questionnaire, establish measurement validity or internal consistency, or support causal conclusions about household livelihood status.

For the benchmark analysis, the identifier variable id was excluded from the predictor set, and community was used as the target variable. After excluding these two columns, the benchmark dataset contained 75 predictor variables**,** one binary target variable, and 282 household observations. The target variable represented two balanced household classes: fishing and non-fishing households. The labels were encoded into binary form for supervised classification, with fishing and non-fishing households represented as the two outcome classes.

The dataset was divided into training, validation, and testing subsets using stratified splitting. This ensured that the balanced class structure was preserved across the data partitions. The approximate split produced 196 training observations, 43 validation observations, and 43 testing observations. Stratified 10-fold cross-validation was then applied during benchmark evaluation to provide a more stable estimate of model performance across the balanced dataset.

The preprocessing workflow was designed for mixed-type household survey data. Numerical variables were treated separately from categorical and ordinal categorical variables. The pipeline included median imputation for numerical variables and most-frequent imputation for categorical variables as general preprocessing safeguards. However, the cleaned dataset used for the benchmark contained no missing values because logical skip-pattern responses had already been recoded into explicit categories during data cleaning. Therefore, the imputation steps did not replace or alter any values in this analysis. Numerical variables were standardized, while categorical and ordinal categorical variables were transformed using one-hot encoding.

Seven supervised classification algorithms were evaluated: Logistic Regression, Random Forest, Support Vector Machine, K-Nearest Neighbors, Decision Tree, Gradient Boosting, and Naive Bayes. Model performance was summarized using accuracy, precision, recall, F1-score, and ROC-AUC. These metrics were used to describe overall classification performance, class-wise prediction quality, and discriminatory ability.

Two benchmark settings were prepared. The first setting used the full predictor set. The second setting used a reduced predictor set after removing occupation-linked or leakage-sensitive variables. The removed variables were work_risk, natural_disaster, piracy, income_beyond_primary, alternative_income, alternative_income_type, own_land_boat_net, income_beyond_season, working_training, and loan_source. These variables were excluded because they may directly or indirectly reflect occupation-specific questionnaire context, including fishing assets, cultivation assets, occupation-related training, workplace risks, or occupation-related loan sources.

The reduced-feature benchmark was used as the final conservative benchmark setting. It retained socioeconomic, education, household, health, food-security, migration, and participation-related variables while excluding variables that could directly reveal livelihood group membership. The final selected benchmark model was a reduced-feature Gradient Boosting classifier. The benchmark outputs were saved in the results/ folder, including benchmark tables, classification reports, confusion matrices, ROC curve files, feature-importance files, final test-set results, and the saved final model.

### 4.7 Machine-learning benchmark results

The benchmark results are summarized in Table 6. The full-feature benchmark produced high classification performance across several models; however, the reduced-feature setting was used as the more conservative benchmark because occupation-linked variables were removed before final model selection.

**Table 6:** Full-feature and reduced-feature machine-learning benchmark comparison.

Model	Accuracy full	F1 full	ROC-AUC full	Accuracy reduced	F1 reduced	ROC-AUC reduced
Gradient Boosting	0.985961	0.985951	0.999014	0.939655	0.939858	0.986054
Random Forest	0.971675	0.971363	1.000000	0.922044	0.919048	0.988061
Support Vector Machine	0.985837	0.986207	0.998980	0.918473	0.914150	0.980510
K-Nearest Neighbors	0.950369	0.948573	0.992449	0.907759	0.903647	0.968469
Logistic Regression	0.978695	0.978809	0.998980	0.900616	0.896785	0.961599
Decision Tree	0.943596	0.943744	0.944048	0.861946	0.862955	0.862381
Naive Bayes	0.670320	0.748527	0.752143	0.599138	0.709935	0.617653

Table 6 reports the benchmark performance of the seven classification models under the full-feature and reduced-feature settings. Because occupation-linked variables were removed from the reduced-feature setting, this setting was retained for the final reproducibility example. Under the reduced-feature setting, Gradient Boosting produced an accuracy of 0.939655, an F1-score of 0.939858, and a ROC-AUC of 0.986054. The remaining model results are reported in Table 6 for completeness and reproducibility, without further substantive interpretation.

The final selected model was the reduced-feature Gradient Boosting classifier. This model was evaluated on an independent test set containing 43 observations. The final test-set performance is summarized in Table 7.

**Table 7:** Final independent test-set result for the selected reduced-feature model.

Model	Feature set	Test accuracy	Test precision	Test recall	Test F1-score	Test ROC-AUC	Test samples
Gradient Boosting	Reduced	0.906977	0.869565	0.952381	0.909091	0.974026	43

The final test-set confusion matrix showed 19 correctly classified fishing households and 20 correctly classified non-fishing households. Three fishing households were classified as non-fishing, and one non-fishing household was classified as fishing. The confusion matrix, ROC curve, classification report, feature-importance outputs, final test-set results, and saved final model are provided in the results/ folder for independent inspection and reproducibility.

Feature-importance outputs for the reduced-feature Gradient Boosting model are also provided in the repository as reproducibility artifacts. These outputs are included to document the benchmark workflow and are not interpreted here as evidence of substantive relationships between individual survey variables and household livelihood status.

Overall, these results should be interpreted as a reproducible classification benchmark rather than as evidence of dataset validity or internal consistency. The reported classification metrics are presented solely as outputs of the reproducible benchmark workflow and are not used to support substantive, inferential, causal, or policy conclusions about the surveyed households. The benchmark outputs are provided as supplementary analytical and reproducibility resources for users who wish to inspect or reuse the workflow. No hypothesis testing, inferential comparison, causal interpretation, or policy conclusion is drawn from the machine-learning results.

### 4.8 Model training setup and computing platform

The machine-learning benchmark workflow was implemented in a reproducible notebook environment using Google Colab. The cleaned dataset, metadata preparation, benchmark evaluation, result-file generation, and final model export were performed using Python-based data analysis and machine-learning libraries.

The main libraries used in the workflow were pandas, numpy, scikit-learn, fastai, matplotlib, joblib, and openpyxl. These libraries were used for data handling, preprocessing, model training, cross-validation, metric calculation, visualization, Excel and CSV file export, and model serialization. Random states were fixed where applicable, mainly using random_state = 42, to support reproducibility of the benchmark outputs.

The final outputs were exported into the results/ folder in CSV, Excel, PNG, PDF, and PKL formats. These outputs include benchmark result tables, full-versus-reduced comparison files, classification reports, confusion matrices, ROC curve files, feature-importance outputs, independent test-set results, and the saved reduced-feature Gradient Boosting model. The complete cleaning and benchmark workflows are provided in 01_data_cleaning.ipynb and 02_ml_benchmark_clean_reproducible.ipynb, respectively.

## Limitations

The dataset is based on household-level survey responses collected from Chamari Union in Singra Upazila, Natore District, Bangladesh. As a result, the findings are specific to this local setting and should not be considered nationally representative of fishing and non-fishing households in Bangladesh. The sample included 282 households, with 141 fishing and 141 non-fishing households selected to allow a balanced comparison between the two groups. This equal split was chosen for comparative purposes and does not reflect the actual proportion of fishing and non-fishing households in the source population. The exact numbers of households in each stratum were not retained in the final study records. Therefore, pooled estimates from the full sample should be interpreted with caution and should not be treated as population-level estimates for Chamari Union without information on the underlying group proportions and appropriate weighting.

The machine-learning results should also be interpreted in light of the relatively small sample size. The benchmark was based on 282 households, while the full-feature setting included 75 predictor variables. Although a reduced-feature setting was also evaluated and occupation-linked variables were removed before the final benchmark, the number of observations remains limited for drawing broad conclusions from the model performance. The reported results should therefore be viewed as a dataset-specific classification benchmark and an example of computational reuse rather than evidence of measurement validity or general performance beyond this sample. Further studies with larger and more diverse household samples would be needed to assess how well the classification results generalize to other settings.

The data were collected through face-to-face interviews using structured questionnaires. As a result, responses may be affected by self-reporting, recall bias, or respondents’ willingness to disclose sensitive information such as income, savings, food scarcity, loans, and welfare access. Most variables are categorical or ordinal categorical because they were derived from predefined questionnaire responses. For example, age was recorded as age groups rather than exact age values. Some questionnaire items with multiple response options were coded into separate variables during data processing. Users should consider these coding choices, variable formats, and the local wetland context when reusing the dataset for statistical, comparative, or computational analysis.

## Ethics Statement

This study involved household-level survey responses from fishing and non-fishing households in the Chalan Beel region, Singra Upazila, Natore District, Bangladesh. The survey was conducted as an academic research activity under departmental supervision. Formal review by an institutional ethics committee or Institutional Review Board (IRB) was not sought, and no formal ethics approval or reference number was issued. The departmental and local administrative permissions obtained for the study related to conducting the academic field survey and should not be interpreted as formal human-subjects ethics approval. Before data collection, all respondents were informed about the purpose of the study, the voluntary nature of their participation, and the academic use of the collected information. Informed consent was obtained from all respondents prior to participation in the survey. The released dataset does not include respondent names, contact information, or direct personal identifiers. During data preparation, personally identifying information was removed, and the id variable was retained only as an anonymized household-level identifier for dataset organization. The dataset is shared for research, teaching, and reproducibility purposes.

## CRediT Author Statement

**Arijit Biswas Anirban**: Conceptualization, Methodology, Data curation, Data cleaning, Software, Formal analysis, Machine-learning benchmark design, Visualization, Writing: original draft, Writing: review and editing.

**Md. Nayem Hossain**: Conceptualization, Questionnaire design, Methodology, Investigation, Field survey coordination, Data collection, Data review, Writing: review and editing.

**Md. Ashik Ali**: Conceptualization, Questionnaire design, Methodology, Investigation, Field survey coordination, Data collection, Data review, Writing: review and editing.

**Sumaiya Khatun**: Data collection, Investigation, Questionnaire support, Data review.

**Istiak Ahammed**: Data collection, Investigation, Questionnaire support, Data review.

**Priyosree Paul Chowdhury**: Data collection, Investigation, Questionnaire support, Data review.

**Md. Zubair Azam**: Data collection, Investigation, Questionnaire support, Data review.

**Nakib Aman**: Supervision, Methodology, Investigation, Data review.

## Declaration of Competing Interest

The authors report no financial or personal conflicts of interest between themselves and others.

## Data Availability

Mendeley DataHousehold Socioeconomic and Livelihood Dataset of Fishermen and Non-Fishermen in Chalan Beel, Bangladesh (Original data)

## Appendix Supplementary materials

Image, application 1
