# Supplementary material for: Dataset on social and economic indicators of fishing and non-fishing households from Natore, Bangladesh
Source: Data Brief. 2026 Nov 3;69:113396. doi: 10.1016/j.dib.2026.113396 (PMC13769514; doi:10.1016/j.dib.2026.113396)
Supplement: Supplementary file 1 [file mmc1.pdf]

Dept. of Computer Science and Engineering
Pabna University of Science and Technology
Pabna-6600, Bangladesh
Phone: +88 025888 44876
Web : www.pust.ac.bd
Email: cse@pust.ac.bd

কম্পিউটার সায়েন্স এন্ড ইঞ্জিনিয়ারিং বিভাগ
পাবনা বিজ্ঞান ও প্রযুক্তি বিশ্ববিদ্যালয়
পাবনা-৬৬০০, বাংলাদেশ
ফোন: +৮৮ ০২৫৮৮৮ ৪৪৮৭৬

Date: 25 January, 2026

TESTIMONIAL

This is to formally certify that the Department of Computer Science and Engineering, Pabna University of Science and Technology (PUST), permitted the following students of the department to conduct a household-level survey for academic research purposes under the research titled *"Dataset on Social and Economic Indicators from Natore, Bangladesh"*.

Permitted students: Arijit Biswas Anirban; Sumaiya Khatun; Istiak Ahammed; Md. Zubair Azam.

The survey was conducted in the Chalan Beel region, Singra Upazila, Natore District, Bangladesh, for collecting household-level demographic, socioeconomic, livelihood, health, food security, and welfare-related information from fishermen and non-fishermen communities. The activity was carried out under the academic supervision of Nakib Aman, Lecturer, Department of Computer Science and Engineering, Pabna University of Science and Technology.

This authority confirms that the research activity was conducted with institutional awareness and departmental permission. The students were instructed to follow standard ethical research practices, including respectful communication with respondents, voluntary participation, informed consent, confidentiality of collected information, and non-disclosure of personal identifiers. The survey activity was conducted for academic and research purposes and did not disrupt normal academic, administrative, or community activities.

This testimonial is issued upon request for academic, research, publication, and journal submission purposes.

Nakib Aman
Lecturer
Department of Computer Science and Engineering
Pabna University of Science and Technology
Pabna-6600, Bangladesh
